# Alpha-Power Pareto distribution: Its properties and applications

**DOI:** 10.1371/journal.pone.0218027

**Published:** 2019-06-12

**Authors:** Shumaila Ihtisham, Alamgir Khalil, Sadaf Manzoor, Sajjad Ahmad Khan, Amjad Ali

**Affiliations:** 1 Department of Statistics, Islamia College, Peshawar, Khyber Pakhtunkhwa, Pakistan; 2 Department of Statistics, University of Peshawar, Peshawar, Khyber Pakhtunkhwa, Pakistan; Universidade Estadual de Maringa, BRAZIL

## Abstract

In Statistical theory, inclusion of an additional parameter to standard distributions is a usual practice. In this study, a new distribution referred to as Alpha-Power Pareto distribution is introduced by including an extra parameter. Several properties of the proposed distribution, including moment generating function, mode, quantiles, entropies, mean residual life function, stochastic orders and order statistics are obtained. Parameters of the proposed distribution have been estimated using maximum likelihood estimation technique. Two real datasets have been considered to examine the usefulness of the proposed distribution. It has been observed that the proposed distribution outperforms different variants of Pareto distribution on the basis of model selection criteria.

## Introduction

For the last few decades, improvement over standard distributions has become a common practice in statistical theory. Usually, an additional parameter is added by using generators or existing distributions are combined to obtain new distributions [1]. The purpose of such modification is to bring more tractability to the classical distributions for useful analysis of complex data structures. [2] and [3] developed a methodology of adding a new parameter in existing distributions. [4] presented an idea of beta generated distributions in which parent distribution is beta while baseline distribution can be the cumulative distribution function (cdf) of any continuous random variable. [5] modified the idea of [4] and replaced beta distribution by Kumaraswamy distribution. Further, [6] proposed the idea of T-X family of continuous distributions in which probability density function (pdf) of beta distribution was replaced by the pdf of any continuous random variable and instead of cdf, a function of cdf satisfying certain conditions was used. [7] provided a detail review on methods of generating univariate continuous distributions.

More recently, [8] presented a new method, called alpha power transformation (APT), for including an extra parameter in continuous distribution. Basically, the idea was introduced to incorporate skewness to the baseline distribution. The alpha power transformation is defined as follows:

Let *F(x)* be the cdf of any continuous random variable *X*, then cdf of *APT* family is given as

$$F_{APT}\left(x\right)=\{\begin{array}{ll} \frac{\alpha^{F\left(x\right)}-1}{\alpha -1} & if\alpha >0,\alpha \neq 1\\ F\left(x\right) & if\alpha =1 \end{array}$$ (1)

The corresponding probability density function is

$$f_{APT}\left(x\right)=\{\begin{array}{ll} \frac{log\alpha }{\alpha -1}\alpha^{F\left(x\right)}f\left(x\right) & if\alpha >0,\alpha \neq 1\\ f\left(x\right) & if\alpha =1 \end{array}$$ (2)

Particularly, the generator was used to transform one parameter exponential distribution into two parameter alpha power exponential distribution. Several properties of the proposed distribution were studied including explicit expressions for survival function, hazard function, quantiles, median, moments, moments generating functions, order statistics, mean residual life function and entropies. Also, the shape behavior of pdf, hazard rate function and survival function were examined. [9] and [1] have successfully used the above generator for transforming two parameters Weibull distribution into three parameters alpha power Weibull distribution. The transformation has been applied by different researchers to obtain alpha power transformed distributions including alpha power transformed generalized exponential distribution [10], alpha power transformed Lindly distribution [11], alpha power transformed extended exponential distribution [12], alpha power transformed inverse Lindly distribution [13] etc.

Pareto distribution is a well-known distribution used to model heavy tailed phenomena [14]. It has many applications in actuarial science, survival analysis, economics, life testing, hydrology, finance, telecommunication, reliability analysis, physics and engineering [15–17]. Pareto distribution is successfully used by [18] for projection of losses in an insurance company, real state and liability experience of hospitals. [16] applied Pareto distribution to model sea clutter intensity returns. [19] used Pareto distribution for investigation of wealth in society. [20] considered generalized form of Pareto distribution to model exceedances over a margin in flood control. Many types of Pareto distribution and its generalization are available in literature. The Pareto distribution of first kind as described by [21] has the cdf as follows:

$$\begin{array}{ll} F\left(x\right)=1-{\left(\frac{k}{x}\right)}^{\beta } & k>0;\beta >0;x\geq k \end{array}$$ (3)

It has two parameters *α* and *k*, where *k* is the lower bound of the data. [18] normalized the data by dividing each observation by the pre-selected lower bound that gives *k* = 1. Eventually, the cdf and pdf of Pareto distribution can be written as

$$\begin{array}{ll} F\left(x\right)=1-x^{-\beta } & x\geq 1,\beta >0 \end{array}$$ (4)

$$\begin{array}{ll} f\left(x;\beta \right)=\frac{\beta }{x^{\beta +1}} & x\geq 1,\beta >0 \end{array}$$ (5)

where *β* is the scale parameter. As the hazard rate function of Pareto distribution is decreasing and has reversed J shaped pdf, it may occasionally be inadequate to fit the data well. Practically, there can be various options for projection of risks and losses, for example, machine life cycle and human mortality has more flexible behavior. That is why researchers proposed various amendment and extensions of the Pareto distribution with different number of parameters [17]. For example, Generalized P [22], Exponentiated P [23,24], Beta P [25], Beta Generalized P [26], Weibull P [27,28], Kumaraswamy P [29], Kumaraswamy Generalized P [30], Exponentiated Weibull P [31], The Burr X-P [17], Exponentiated Generalized P [14].

The aim of this study is to propose a new and more flexible distribution, which, we call Alpha Power Pareto (APP) distribution, by introducing an additional parameter to Basic Pareto distribution, to obtain an adequate fit. Numerous properties of the APP distribution are studied in the following section along with more attractive expressions for quantile function, median, mode, moments, order statistics, mean residual life function and stress strength parameter. Lemma 1 and 2 contains expressions for stochastic ordering, Shannon and Renyi entropies respectively. The next section provides method of maximum likelihood estimation of parameters in addition to simulation studies. Two real data applications are used to check the effectiveness of the proposed model. Conclusions are provided in the last section.

## Alpha Power Pareto (APP) distribution

Random variable *X* is said to have an APP distribution if its pdf is of the form

$$f_{APP}\left(x\right)=\{\begin{array}{ll} \frac{\beta log\alpha }{\alpha -1}\alpha^{1-x^{-\beta }}x^{-\beta -1} & \alpha \neq 1\\ f\left(x\right) & \alpha =1 \end{array}$$ (6)

and 0 otherwise. By setting *x*^-*β*^ = *z* in Eq (6), it can be easily verified that

$$\int_{1}^{\infty }f_{APP}(x)=1$$

The corresponding cdf of APP distribution is

$$F_{APP}\left(x\right)=\{\begin{array}{ll} \frac{\alpha^{1-x^{-\beta }}-1}{\alpha -1} & \alpha \neq 1\\ 1-x^{-\beta } & \alpha =1 \end{array}$$ (7)

The survival (reliability) function and hazard rate function are obtained, respectively, as follows:

$$S_{APP}\left(x\right)=\{\begin{array}{ll} \frac{\alpha }{\alpha -1}\left(1-\alpha^{-x^{-\beta }}\right) & \alpha \neq 1\\ x^{-\beta } & \alpha =1 \end{array}$$ (8)

$$h_{APP}\left(x\right)=\{\begin{array}{ll} \frac{\beta log\alpha }{1-\alpha^{-x^{-\beta }}}\alpha^{-x^{-\beta }}x^{-\beta -1} & \alpha \neq 1\\ \frac{\beta }{x} & \alpha =1 \end{array}$$ (9)

Henceforth, a random variable *X* that follows the distribution in (6) is symbolized by *X~APP(α*, *β)*.

**Fig 1:**
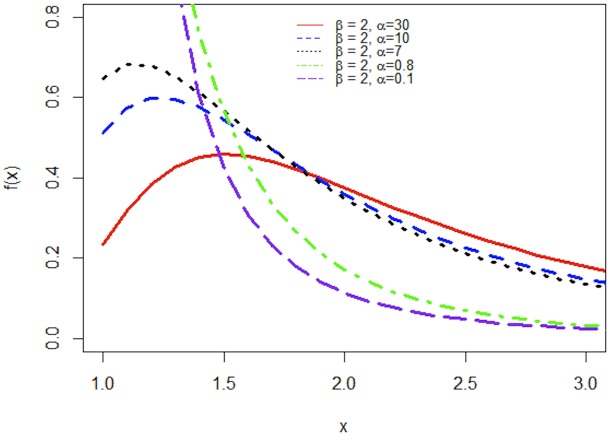
The PDF of APP distribution for various values of α and fixed β.

**Fig 2:**
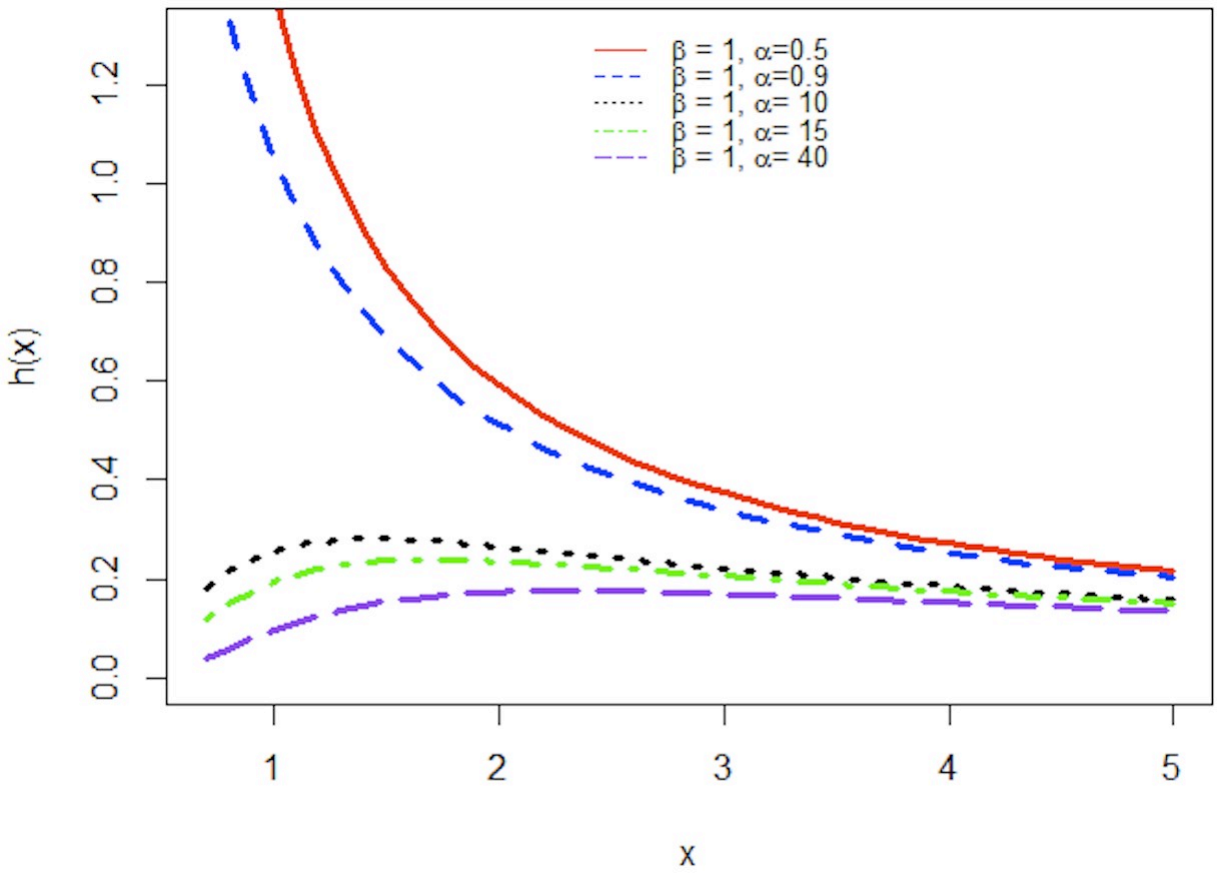
Increasing, decreasing shapes of hazard function of APP distribution.

Figs 1 and 2 demonstrate the graphs of pdf and hazard function of APP distribution for different values of *α* when *β* is fixed. Clearly, the pdf of APP distribution is decreasing function for *α* < 1 and uni-modal and positively skewed for *α* < 1.

### Quantile function

Quantile function is defined as an inverse of the distribution function. Consider the identity

$$F(X)=U\Rightarrow X=F^{-1}(U)$$

where *U* follows standard Uniform distribution. The *p*^*th*^ quantile of APP distribution is given by

$$x_{p}={\left[\frac{log(\;\alpha /(p(\alpha -1)+1))}{log\alpha }\right]}^{-1/\beta }$$ (10)

Median of APP distribution can be obtained by putting *p = 1/2*, that is,

$$x_{1/2}={\left[\frac{\left(log\frac{2\alpha }{\alpha +1}\right)}{log\alpha }\right]}^{-1/\beta }$$ (11)

### Mode

The mode of the distribution can be found by solving the following equation

$$\frac{d}{dx}f_{APP}(x)=0$$

By taking the derivative of Eq (6) and equating it to zero and solving for *x*, mode becomes

$$x={\left[\frac{\beta +1}{\beta log\alpha }\right]}^{-1/\beta }$$ (12)

In Table 1 mode of the APP distribution is calculated for different choices of *α* and *β*. These results can be verified through Fig 1.

**Table 1:** Mode for different choices of *α* and *β*.

*β*	*α*	Mode
2	40	1.568
	30	1.505
	20	1.413
	10	1.238
	5	1.035

### Moments

The moment generating function of APP distribution is given by

$$M_{x}(t)=E[e^{tx}]=\int_{1}^{\infty }e^{tx}\frac{\beta log\alpha }{\alpha -1}\alpha^{1-x^{-\beta }}x^{-\beta -1}dx$$ (13)

by substituting *x*^-*β*^ = *z* and the following series representation

$$e^{tx}=\sum_{r=0}^{\infty }\frac{t^{r}x^{r}}{r!}$$

$$\alpha^{-z}=\sum_{k=0}^{\infty }\frac{{\left(-log\alpha \right)}^{k}}{k!}z^{k},$$ (14)

it can be easily verified that

$$M_{x}(t)=\frac{\alpha \beta }{1-\alpha }\sum_{k=0}^{\infty }\sum_{j=0}^{\infty }\frac{{\left(-log\alpha \right)}^{k+1}\;t^{j}}{k!j!(k\beta -j+\beta )}$$ (15)

by taking derivative of Eq (15) and putting *t = 0*,*1*,*2…r*, *E(X)*, *E(X*^*2*^ *)*,*…E(X*^*r*^*)* of APP distribution are obtained as

$$E(X)=\frac{\alpha \beta }{\left(1-\alpha \right)}\sum_{k=0}^{\infty }\frac{{\left(-log\alpha \right)}^{k+1}}{k!}[\frac{1\;}{\left(k\beta +\beta -1\right)}]$$ (16)

$$E(X^{2})=\frac{\alpha \beta }{\left(1-\alpha \right)}\sum_{k=0}^{\infty }\frac{{\left(-log\alpha \right)}^{k+1}}{k!}[\frac{2.1\;}{\left(k\beta +\beta -2\right)}]$$ (17)

$$E(X^{r})=\frac{\alpha \beta }{\left(1-\alpha \right)}\sum_{k=0}^{\infty }\frac{{\left(-log\alpha \right)}^{k+1}}{k!}[\frac{r!}{\left(k\beta +\beta -r\right)}]$$ (18)

### Mean residual life function

Assuming that *X* is a continuous random variable with survival function given in Eq (8), the mean residual life function is defined as the expected additional lifetime that a component has survived until time *t*. The mean residual life function, say, *μ(t)* is given by

$$\mu (t)=\frac{1}{P(X>t)}\int_{t}^{\infty }P(X>x)dx\;,\;t\geq 0$$

$$\mu (t)=\frac{1}{S(t)}(E(t)-\int_{0}^{t}xf(x)dx)-t\;,\;t\geq 0$$ (19)

where

$$\int_{0}^{t}xf(x)dx=\frac{\beta \alpha log\alpha }{\alpha -1}\sum_{k=0}^{\infty }\frac{{\left(-log\alpha \right)}^{k}}{k!(k\beta +\beta -1)}(-t^{-(k\beta +\beta -1)})$$ (20)

Substituting Eqs (8), (16) and (20) in Eq (19), μ(t) can be written as

$$\mu (t)=\frac{\beta \alpha log\alpha }{\left(1-\alpha^{-t^{-\beta }}\right)}\sum_{k=0}^{\infty }\frac{{\left(-log\alpha \right)}^{k}}{k!(k\beta +\beta -1)}({1+t}^{-(k\beta +\beta -1)})-t$$ (21)

### Stochastic ordering

Stochastic ordering plays a significant role for assessing the comparative behavior of continuous random variable. It is known that if a distribution has likelihood ratio (*lr*) ordering, then it possesses the same ordering in hazard rate (*hr*) and distribution (*st*). It is also known that if a family of distribution has likelihood ratio ordering, then there exists a uniformly most powerful test [32].

**Lemma 1:** Let *X*_1_*~APP(α*_1_, *β)* and *X*_2_*~APP(α*_2_, *β)* be two independent random variables. If *α*_1_ < *α*_2_ then

$$X_{1}\leq_{lr}{\;X}_{2}\;\forall \;X$$

**Proof:** Likelihood ratio is given by

$$\frac{f_{X_{1}}(x)}{f_{X_{2}}(x)}=(\frac{log\alpha_{1}}{log\alpha_{2}})(\frac{\alpha_{2}-1}{\alpha_{1}-1}){\left(\frac{\alpha_{1}}{\alpha_{2}}\right)}^{1-x^{-\beta }}$$

$$\frac{d}{dx}(log\frac{f_{X_{1}}(x)}{f_{X_{2}}(x)})=log(\frac{\alpha_{1}}{\alpha_{2}})(\beta x^{-\beta -1})<0\;,\;if\;\alpha_{1}<\alpha_{2},\forall \;x>0$$

Hence, for

$$\alpha_{1}<\alpha_{2},\;X_{1}\leq_{lr}{\;X}_{2}$$

for all *x*, it also follows that

$$X_{1}\leq_{hr}{\;X}_{2}\Rightarrow X_{1}\leq_{st}{\;X}_{2}$$

### Order statistics

Let *X*_*1*_, *X*_*2*_, *X*_*3*_, …, *X*_*n*_ be a random sample of size *n* from APP distribution and let *Y*_*i*:*n*_ denote the *i*^*th*^ order statistics, then the pdf of *Y*_*i*:*n*_ is given by

$$f_{i:n}(y)=\frac{n!}{(i-1)!(n-i)!}fx(y)\;{\left[Fx(y)\right]}^{i-1}\;[{1-Fx(y)]}^{n-i}$$ (22)

substituting the pdf and cdf of APP distribution in (22), we get the pdf of *i*^*th*^ order statistics for *y>1* as

$$f_{i:n}(y)=\frac{n!\;\beta log\alpha }{\left(i-1)!(n-i)!(\alpha -1\right)}\alpha^{1-y^{-\beta }+n-i}{\;y}^{-\beta -1}{\left(\alpha^{1-y^{-\beta }}-1\right)}^{i-1}{\left(1-\alpha^{-y^{-\beta }}\right)}^{n-i}$$ (23)

by putting *i = 1*, we get first order statistics as

$$f(y_{1})=\frac{n\beta log\alpha }{{\left(\alpha -1\right)}^{n}}\alpha^{n-y^{-\beta }}y^{-\beta -1}{{(1-\alpha }^{-y^{-\beta }})}^{n-1}$$ (24)

by putting *i = n* we get *n*^*th*^ order statistics as

$$f(y_{n})=\frac{n\beta log\alpha }{{\left(\alpha -1\right)}^{n}}\alpha^{1-y^{-\beta }}y^{-\beta -1}{{(\alpha }^{1-y^{-\beta }}-1)}^{n-1}$$ (25)

### Stress-strength parameter

Suppose *X*_*1*_ and *X*_*2*_ be two continuous and independent random variables, where *X*_1_*~APP(α*_1_, *β)* and *X*_2_*~APP(α*_2_, *β)* then the stress strength parameter, say *S*, is defined as

$$S=\int_{-\infty }^{\infty }f_{1}(x)F_{2}(x)dx$$

using the pdf and cdf of APP distribution, stress strength parameter *S*, can be obtained as

$$S=\frac{\alpha_{1}\beta log\alpha_{1}}{\alpha_{1}-1}\int_{1}^{\infty }\alpha_{1}^{{-x}^{-\beta }}x^{-\beta -1}(\alpha_{2}^{1-x^{-\beta }}-1)dx$$ (26)

The use of (14) in Eq (26) yields

$$S=\frac{\alpha_{1}log\alpha_{1}}{\left(\alpha_{1}-1)(\alpha_{2}-1\right)}\sum_{k=0}^{\infty }\frac{{\left(-log\alpha_{1}\right)}^{k}}{k!}[\alpha_{2}\sum_{m=0}^{\infty }\frac{{\left(-log\alpha_{2}\right)}^{m}}{m!(k+m+1)}-\frac{1}{k+1}]$$ (27)

**Lemma 2:** Shannon and Renyi entropy for random variable *X* that follows Alpha Power Pareto distribution is as follows

$${SE}_{x}=log\frac{\alpha -1}{\alpha \beta \;log\alpha }+\frac{\alpha }{\alpha -1}\sum_{k=0}^{\infty }\frac{({-log\alpha )}^{k+1}}{k!}[\frac{\left(-log\alpha \right)}{k+2}-\frac{\beta +1}{{\beta (k+1)}^{2}}]$$ (28)

$${RE}_{x}=\frac{\rho }{\;1-\rho }log[\frac{\beta \alpha log\alpha }{\alpha -1}]+\frac{1}{1-\rho }log\sum_{k=0}^{\infty }\frac{{\left(-log\alpha \right)}^{k\;}\rho^{k}}{k!(\rho \beta +\rho -1+k\beta )}$$ (29)

**Proof**:

For APP distribution, the Shannon and Renyi entropies are given respectively as

$$E[-log(f(x)]=\int_{1}^{\infty }log(f(x))f(x)dx$$

$$\frac{1}{1-\rho }\text{log}\int_{-\infty }^{\infty }{f(x)}^{\rho \;}dx=\frac{1}{1-\rho }\int_{1}^{\infty }{\left(\frac{\beta log\alpha }{\alpha -1}\alpha^{1-x^{-\beta }}x^{-\beta -1}\right)}^{\rho }dx$$

the results can be obtained easily by using Eq (14).

## Parameters estimation

### Maximum likelihood estimation

Let *X*_*1*_, *X*_*2*_, *X*_*3*_, …, *X*_*n*_ be a random sample from APP (*α*_*1*_, *β*) then the likelihood function is given by

$${l(\alpha ,\beta )=\beta^{n}{(\frac{log\alpha }{\alpha -1})\;}^{n}\alpha }^{n-\sum {x_{i}}^{-\beta }}\prod_{i=1}^{n}x_{i}^{-\beta -1}$$ (30)

taking logarithm, Eq (32) becomes

$$logl(\alpha ,\beta )=nlog\beta +nlog(\frac{log\alpha }{\alpha -1})+(n-\sum x_{i}^{-\beta })log\alpha +(-\beta -1)\sum logx_{i}$$

taking derivative of the above equation with respect to *α* and *β* and equating to zero, the following two normal equations are obtained

$$\frac{\partial logl(\alpha ,\beta )}{\partial \alpha }=\frac{n(\alpha -1-\alpha log\alpha )}{\alpha (\alpha -1)log\alpha }+\frac{n-{\sum x}_{i}^{-\beta }}{\alpha }=0$$ (31)

$$\frac{\partial logl(\alpha ,\beta )}{\partial \beta }=\frac{n}{\beta }+{\sum x}_{i}^{-\beta }logx_{i}log\alpha -\sum logx_{i}=0$$ (32)

by solving (31) and (32) simultaneously, MLE of *α* and *β* can be obtained. Standard algorithm like Newton Raphson method or Bisection method can be used to solve these nonlinear equations. It is well known that MLEs are asymptotically normally distributed i.e, $\sqrt{n}(\hat{\alpha }-\alpha ,\hat{\beta }-\beta )\sim N_{2}(0,\Sigma )$ where Σ is variance covariance matrix and can be obtained by inverting observed Fisher information matrix *F* as given below

$$F=\left[\begin{array}{cc} \frac{\partial^{2}logl}{\partial \alpha^{2}} & \frac{\partial^{2}logl}{\partial \alpha \partial \beta }\\ \frac{\partial^{2}logl}{\partial \alpha \partial \beta } & \frac{\partial^{2}logl}{\partial \beta^{2}} \end{array}\right]$$

taking second derivative of Eqs (31) and (32) w.r.t *α* and *β*

$$\frac{\partial^{2}logl}{\partial \alpha^{2}}=\frac{n}{{\left(\alpha -1\right)}^{2}}-\frac{nlog\alpha +n}{\alpha^{2}l{og}^{2}\alpha }-\frac{n-\sum x_{i}^{-\beta }}{\alpha^{2}}$$ (33)

$$\frac{\partial^{2}logl}{\partial \alpha \partial \beta }=\frac{\sum x_{i}^{-\beta }logx_{i}}{\alpha }$$ (34)

$$\frac{\partial^{2}logl}{\partial \beta^{2}}=-\frac{n}{\beta^{2}}-log\alpha \sum x_{i}^{-\beta }{\left(logx_{i}\right)}^{2}$$ (35)

Asymptotic (1 − *ζ*)100% confidence intervals for parameters can be obtained as

$$\hat{\alpha }\pm Z_{\zeta /2}\sqrt{\Sigma_{11}}$$

$$\hat{\beta }\pm Z_{\zeta /2}\sqrt{\Sigma_{22}}$$

where *Z*_*ζ*_ is the upper ζ*^th^* percentile of the standard normal distribution.

### Simulations study

Simulation study has been performed for average MLEs, Mean Square Error (MSE) and bias. W = 1000 samples of size n = 50, 80, 100 and 120 were produced form APP distribution. Random numbers were generated by the following expression

$$X={\left[\frac{log\;(\alpha /(U(\alpha -1)+1))}{log\alpha }\right]}^{-1/\beta }$$

where *U* is uniform random numbers with parameter *[0*, *1]*. Bias and MSE are calculated by

$$Bias=\frac{1}{W}\sum_{1=1}^{w}\left(\hat{b_{i}}-b\right)$$

$$MSE=\frac{1}{W}{\sum_{1=1}^{w}\left(\hat{b_{i}}-b\right)}^{2}$$

where *b* = (*α*, *β*). Simulations results were obtained for different combinations of *α* and *β*. The average values of MSEs and Bias are displayed in Table 2. It can be illustrated clearly that these estimates are reasonably consistent and approaches to the true values of parameters as sample size increases. Furthermore, with increasing sample size the MSEs and Bias decrease for all parameter combinations. Therefore, it has been concluded that MLE process performs well in estimating the parameters of APP distribution.

**Table 2:** Average values of MLE, corresponding MSE and Bias.

Parameter	N	Mean($\hat{\alpha }$)	Mean($\hat{\beta }$)	MSE($\hat{\alpha }$)	MSE($\hat{\beta }$)	Bias($\hat{\alpha }$)	Bias($\hat{\beta }$)
α = 1.5 β = 2	50	2.362798	2.11534	4.56759	0.2688502	0.8627983	0.11534
	80	2.071618	2.055127	2.747875	0.1810486	0.5716183	0.055127
	100	1.903387	2.043762	1.766545	0.1305861	0.4033868	0.043762
	120	1.831531	2.04308	1.310119	0.1112204	0.3315312	0.043079
	200	1.695633	2.019918	0.6636205	0.06590826	0.1956325	0.019917
α = 0.5 β = 2	50	1.026814	2.214347	1.715091	0.5716235	0.526819	0.2143466
	80	0.736057	2.068304	0.5273031	0.3381399	0.2360573	0.0683033
	100	0.732957	2.103237	0.3562664	0.290025	0.2329578	0.1032366
	120	0.683433	2.09421	0.2801175	0.3199995	0.1834335	0.0942097
	200	0.595542	2.037622	0.1361035	0.1468591	0.0955428	0.0376215
α = 1.5 β = 2	50	2.91755	2.073453	5.899037	0.2123235	0.9175495	0.07345263
	80	2.622482	2.057547	3.544905	0.1387262	0.62284816	0.05754672
	100	2.442603	2.031565	2.440172	0.111905	0.4426029	0.03156455
	120	2.379592	2.02482	1.913967	0.09221928	0.3795922	0.02481982
	200	2.259941	2.024696	1.06941	0.05798964	0.2599414	0.02469612
α = 5 β = 2	50	5.710379	2.020932	9.390076	0.1045967	0.710379	0.02093233
	80	5.5189	2.018883	5.91201	0.06479451	0.3518904	0.01888338
	100	5.205472	1.993133	3.631121	0.05078997	0.2054723	0.0068665
	120	5.101856	1.995896	2.943594	0.04143445	0.1018556	0.0041041
	200	5.098387	1.996232	2.914719	0.03991468	0.0983866	0.0037680

### Applications

Two data sets have been analyzed to demonstrate the performance of the proposed model. The first data set consists of 40 wind related catastrophes used by [33]. It includes claims of $2,000,000. The sorted values, observed in millions are as follows.

**Table:** 

2	2	2	2	2	2	2	2	2	2	2	2	3	3	3	4	4	4	5
5	5	6	6	6	6	8	8	9	15	17	22	23	24	24	25	27	32	43

The second data set consists of survival time (in weeks) of 33 acute myelogenous leukaemia patients. The data has been analysed by [17, 34]. The data values are as follows.

**Table:** 

65	156	100	134	16	108	121	4	39	143	56
26	22	1	1	5	65	56	65	17	7	16
22	3	4	2	3	8	4	3	30	4	43

The fit of the proposed APP distribution is compared with several other competitive models namely Basic Pareto, Pareto distribution by [35], Genaralized Pareto distibution by [22], Kumaraswamy Pareto distribution by [29], Exponentiated Generalized Pareto Distribution by [14] and Inverse Pareto distribution [36] with the following pdfs.

- Basic Pareto Distribution (BP)
  $$\begin{array}{cc} f\left(x\right)=\frac{\beta }{x^{\beta +1}} & \beta >0,\;X\geq 1 \end{array}$$
- Pareto Distribution (PD)
  $$\begin{array}{cc} f\left(x\right)=\frac{\sigma \beta^{\sigma }}{{\left(x+\beta \right)}^{\sigma +1}} & \sigma ,\;\beta >0,\;X\geq 0 \end{array}$$
- Generalized Pareto Distribution (GPD)
  $$f(x)=\frac{1}{\delta }{\left(1+\frac{\xi x}{\delta }\right)}^{-\frac{1}{\xi }-1}\xi \neq 0,\;X\geq 0,\delta >0$$
- Kumaraswamy Pareto Distibution (KPD)
  $$\begin{array}{cc} f\left(x\right)=\frac{abk\beta^{k}}{x^{k+1}}{\left[1-{\left(\frac{\beta }{x}\right)}^{k}\right]}^{a-1}{\left[1-{\left\{1-{\left(\frac{\beta }{x}\right)}^{k}\right\}}^{a}\right]}^{b-1} & x\geq \beta ,\;a,b,k>0 \end{array}$$
- Exponentiated Generalized Pareto Distribution (ExGPD)
  $$\begin{array}{cc} f\left(x\right)=\frac{e^{x}}{\delta }{\left(1+\frac{\xi e^{x}}{\delta }\right)}^{-\frac{1}{\xi }-1} & \xi \neq 0,\;-\infty \leq X\leq \infty ,\delta >0 \end{array}$$
- Inverse Pareto Distribution (IPD)
  $$\begin{array}{cc} f\left(x\right)=\frac{\alpha \beta x^{\alpha -1}}{{\left(\beta +x\right)}^{\alpha +1}} & X>0,\;\alpha ,\beta >0 \end{array}$$

The goodness of fit test is applied, using AdequacyModel package of R software, to check the performance of APP distribution and several other versions of Pareto distribution discussed above. Goodness of fit criteria include the result of Akaike’s Information Criteria (AIC), Consistent Akaike’s Information Criteria (CAIC), Bayesian Information Criterion (BIC), Hannan-Quinn Information Criteria (HQIC), *-ln$\left(\hat{\theta }\right)$* along with the result of Kulmogrov-Smirnov test (KS) and its p value as shown in Tables 3 and 4. In general, if the values of all the above criteria are smaller and p value is greater, the model is considered as good fit.

**Table 3:** Goodness of fit result for data set 1.

Distribution	MLE		AIC	CAIC	BIC	HQIC	-ln($\hat{\theta })$	KS	p-value
BP	0.595		251.61	251.61	253.16	252.10	124.7	0.22	0.0502
GPD	0.1655	7.42	251.22	251.55	254.55	252.42	122.6	0.21	0.0600
ExGPD	7.745	21.04	253.22	253.22	256.21	254.07	124.4	0.22	0.0522
IPD	0.390	10.30	242.27	242.59	245.59	243.45	119.1	0.16	0.2097
**APP**	**1.223**	**56.16**	**235.26**	**235.59**	**238.58**	**236.45**	**115.6**	**0.16**	**0.2497**

**Table 4:** Goodness of fit result for data set 2.

Distribution	MLE				AIC	CAIC	BIC	HQIC	-ln($\hat{\theta })$	KS	p-value
BP	0.353				323.41	323.54	324.91	323.91	160.70	0.23	0.059
PD	0.802		9.76		317.14	317.54	320.13	318.14	156.56	0.15	0.402
KPD	3.71	3.91	0.27	0.37	318.16	319.59	324.15	320.18	155.80	0.15	0.406
ExGPD	36.62		15.93		317.74	318.15	320.74	318.75	156.87	0.18	0.203
**APP**	**0.102**		**37.58**		**314.64**	**315.04**	**317.63**	**315.65**	**155.32**	**0.15**	**0.409**

**Fig 3:**
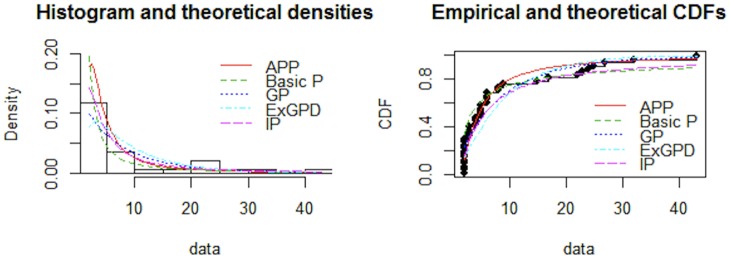
Comparison between fitted distributions for dataset 1.

**Fig 4:**
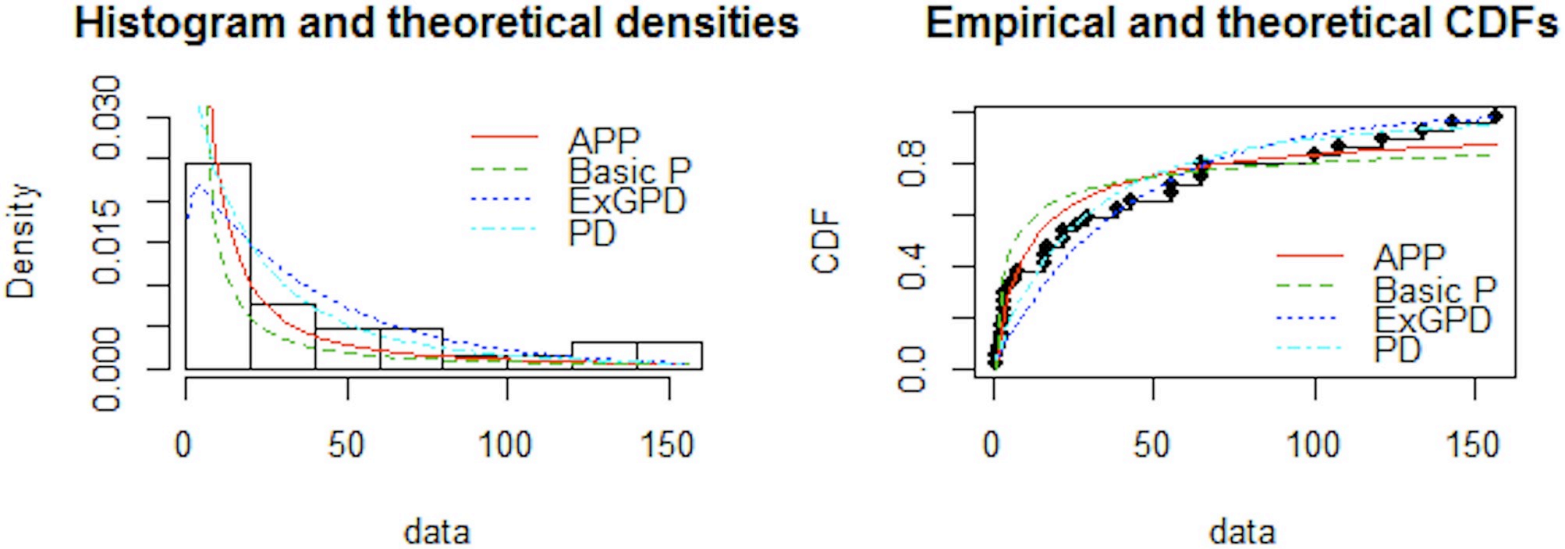
Comparison between fitted distributions for dataset 2.

**Fig 5:**
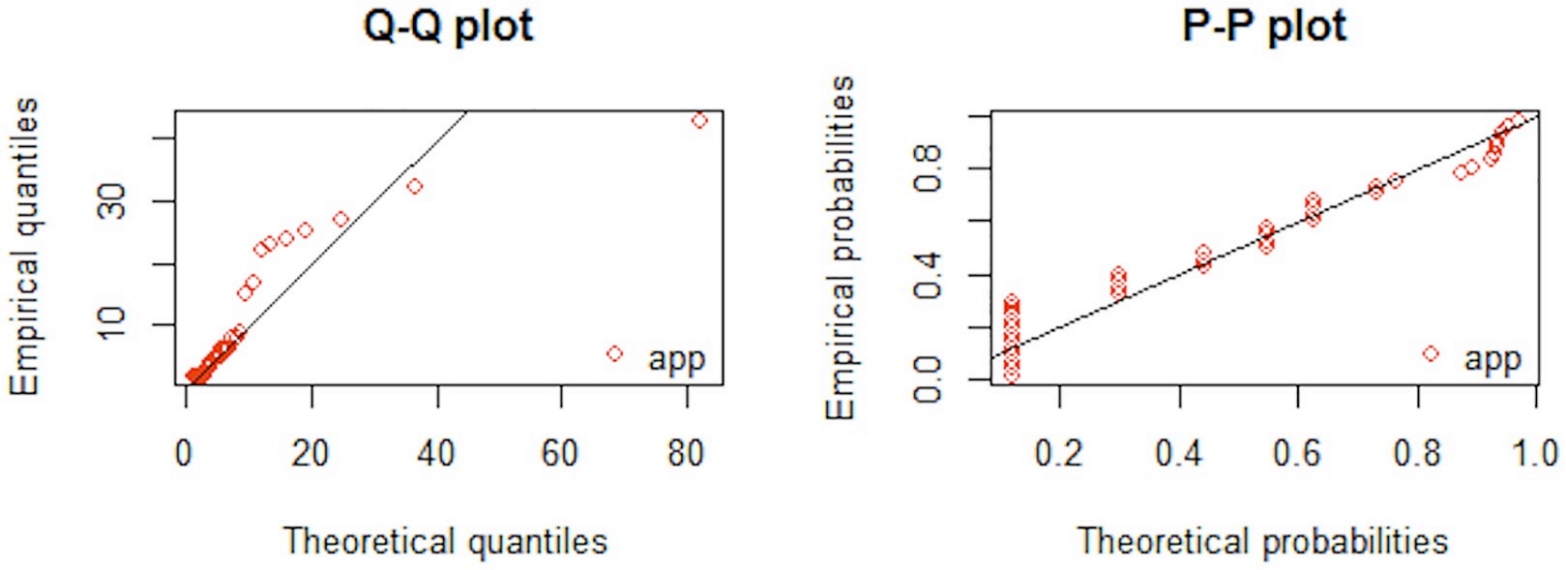
QQ-plot and PP-plot of APP distribution for dataset 1.

**Fig 6:**
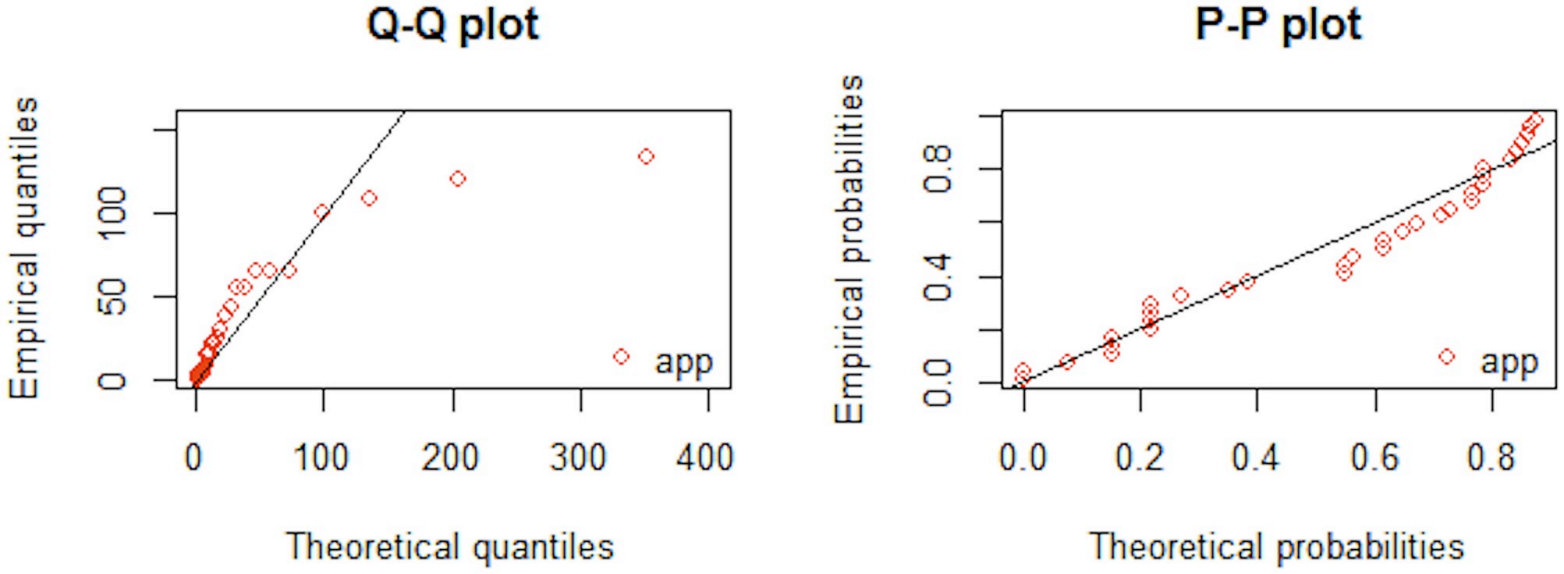
QQ-plot and PP-plot of APP distribution for dataset 2.

From the results provided in Tables 3 and 4 it is evident that AIC, CAIC, BIC, HQIC and -log-likelihood are lower for APP distribution as compared to the other fitted distributions. Promising performance of the proposed distribution is visible from Figs 3 and 4. Figs 5 and 6, QQ-plot and PP-plot is provided. Apparently, some of the values of QQ-plot depart from the fitted line, but actually, it is an expected behavior of a heavy tailed distributions [37].

## Conclusion

The new distribution, termed as APP distribution, is introduced using alpha power transformation. Mainly, the transformation is applied for adding skewness to a family of distribution functions. Different properties of the distribution have been derived including moment generating function, order statistics, stress strength parameter, mean residual life function, mode, stochastic ordering and expressions for entropies. Maximum likelihood estimation procedure has been used to provide parameter estimates of the unknown parameters. The proposed distribution has been applied to two real datasets, which indicates its better performance as compared to other variants of Pareto distributions.

## Supporting information

S1 File Data Set 1. (DOCX)

S2 File Data Set 2. (DOCX)
