# Supplementary material for: Alpha-Power Pareto distribution: Its properties and applications
Source: PLoS One. 2019 Jun 12;14(6):e0218027. doi: 10.1371/journal.pone.0218027 (PMC6561572; doi:10.1371/journal.pone.0218027)
Supplement: S1 File — (DOCX) [file pone.0218027.s001.docx]

S1 File: Data Set 1

The first data set consists of 40 losses that occurred in 1977 due to wind-related catastrophes.

2 2 2 2 2 2 2 2 2 2 2 2 3 3 3 4 4 4 5

5 5 6 6 6 6 8 8 9 15 17 22 23 24 24 25 27 32 43

Source

Hogg, R. and Klugman, S.A. Loss Distributions. New York: Wiley; 1984.
