# Supplementary material for: Alpha-Power Pareto distribution: Its properties and applications
Source: PLoS One. 2019 Jun 12;14(6):e0218027. doi: 10.1371/journal.pone.0218027 (PMC6561572; doi:10.1371/journal.pone.0218027)
Supplement: S2 File — (DOCX) [file pone.0218027.s002.docx]

S1 File: Data Set 2

Survival time (in weeks) of 33 acute myelogenous leukaemia patients is given below.

65 156 100 134 16 108 121 4 39 143 56

26 22 1 1 5 65 56 65 17 7 16

22 3 4 2 3 8 4 3 30 4 43

Source

Korkmaz M, Altun E, Yousof H, Afify A, Nadarajah S. The Burr X Pareto Distribution: Properties, Applications and VaR Estimation. Journal of Risk and Financial Management. 2018; 11(1):1.

Mead ME, Afify AZ, Hamedani GG, Ghosh I. The beta exponential Fréchet distribution with applications. Austrian Journal of Statistics. 2017; 46(1):41-63.
